# The Nuts and Bolts of PIN Auxin Efflux Carriers

**DOI:** 10.3389/fpls.2019.00985

**Published:** 2019-07-31

**Authors:** Marta Zwiewka, Veronika Bilanovičová, Yewubnesh Wendimu Seifu, Tomasz Nodzyński

**Affiliations:** Mendel Centre for Plant Genomics and Proteomics, Central European Institute of Technology (CEITEC), Masaryk University, Brno, Czechia

**Keywords:** PIN efflux carriers, protein domains, sequence motifs, auxin transport, subcellular trafficking

## Abstract

The plant-specific proteins named PIN-FORMED (PIN) efflux carriers facilitate the direction of auxin flow and thus play a vital role in the establishment of local auxin maxima within plant tissues that subsequently guide plant ontogenesis. They are membrane integral proteins with two hydrophobic regions consisting of alpha-helices linked with a hydrophilic loop, which is usually longer for the plasma membrane-localized PINs. The hydrophilic loop harbors molecular cues important for the subcellular localization and thus auxin efflux function of those transporters. The three-dimensional structure of PIN has not been solved yet. However, there are scattered but substantial data concerning the functional characterization of amino acid strings that constitute these carriers. These sequences include motifs vital for vesicular trafficking, residues regulating membrane diffusion, cellular polar localization, and activity of PINs. Here, we summarize those bits of information striving to provide a reference to structural motifs that have been investigated experimentally hoping to stimulate the efforts toward unraveling of PIN structure-function connections.

## Introduction

Auxin is an important regulator of plant development. This signaling molecule provides instructive cues through its accumulation patterns guiding the development from early embryogenesis (Robert et al., 2018) and throughout the entire plant ontogenesis (Vanneste and Friml, 2009), orchestrating the establishment of embryonic apical-basal polarity (Friml et al., 2003), root patterning (Friml et al., 2002a; Ditengou et al., 2008), root system architecture (Lavenus et al., 2016), organogenesis and organ positioning (Heisler et al., 2005; Pahari et al., 2014), and cell differentiation (Marhava et al., 2018). Directional transport of this phytohormone is also involved in responses of plants to critically important environmental stimuli such as gravity (Chen et al., 1998; Luschnig et al., 1998; Kleine-Vehn et al., 2008; Rahman et al., 2010; Rakusová et al., 2011, 2016; Leitner et al., 2012b) and light (Ding et al., 2011; Haga and Sakai, 2012; Zhang et al., 2013).

It has been well established that auxin (with indole-3-acetic acid – IAA – being the most abundant form) exerts its action through the de-repression of numerous auxin-responsive genes (Guilfoyle et al., 1998; Schenck et al., 2010). The main components of auxin signaling machinery encompass proteins from three families: the F-box TRANSPORT INHIBITOR RESPONSE 1/AUXIN SIGNALING F-BOX PROTEIN (TIR1/AFB) auxin co-receptors, the Auxin/INDOLE-3-ACETIC ACID (Aux/IAA) transcriptional repressors, and the AUXIN RESPONSE FACTOR (ARF) transcription factors. Auxin enhances the interaction between TIR1/AFB and Aux/IAA proteins, leading to a proteasome-dependent degradation of Aux/IAAs and a subsequent release of ARF repression (Salehin et al., 2015; Lavy and Estelle, 2016). Auxin is synthesized in cotyledons, young leaves, and other growing tissues, where development coordination is needed, including expanding leaves (Ljung et al., 2001), roots (Ljung et al., 2005), and reproductive organs (Robert et al., 2015; Lv et al., 2019). IAA is structurally related to the amino acid tryptophan and is synthesized through both enzymatic tryptophan-dependent and independent pathways (Ruiz Rosquete et al., 2011; Kasahara, 2016).

Beside localized synthesis or enzymatic modification, the abundance of active IAA is regulated by auxin carriers. Developmentally crucial polar auxin transport (PAT) is established and maintained by the combined action of auxin transporters from at least three families: AUXIN1-RESISTANT1 (AUX1)/LIKE AUX1 (hereafter AUX1/LAX), PIN-FORMED proteins (hereafter PINs), and members of the B subfamily of ATP binding cassette (ABC) transporters (hereafter ABCBs) (Geisler et al., 2014).

The AUX1/LAXs are part of the amino acid permease superfamily (Bennett et al., 1996) and function as auxin-proton co-transporters (Singh et al., 2018). The variable N- and C-termini of AUX1 are positioned in the cytoplasm and apoplast, respectively and the experiments indicate that the protein has 11 transmembrane domains (Swarup et al., 2004). Similar to PINs, sequence identity to a protein for which AUX1 structure has been solved is so low that homology modeling is unrealistic at present. Nevertheless, the topology predictions allow some comparison to mammalian co-transporter proteins (Fowler et al., 2015). The membrane-spanning helices are likely to drive an alternating-access mechanism (Jardetzky, 1996). In the proposed mode of action, when IAA and protons bind to an outward-open conformation, there is a change in helix arrangement giving rise to an inward-open conformation from which the bound substrates dissociate as the protons dissipate in the proton-poor cytoplasm. However, such predictions require crystallographic validation for AUX1/LAXs (Singh et al., 2018).

Unlike PINs and AUX1/LAX proteins, a subgroup of ABCBs act as primary active auxin pumps that is able to transport against steep auxin gradients (Geisler et al., 2016). Those ATP-binding cassette transporters share a common architecture consisting of two transmembrane domains (TMDs) and two cytosolic nucleotide-binding domains (NBDs). Models derived from high-resolution crystal structures of bacterial ABC transporters have provided important insights into the structure of plant ABCs (Bailly et al., 2012). In the commonly accepted ABC transport mechanism, helices from each TMD participate in substrate binding and form the translocation pathway for it, while both NBDs transmit the necessary ATP-dependent energy to perform a complete transport cycle (Lefèvre and Boutry, 2018). Predictions suggest that substrate specificity in plant ABCBs is determined primarily by the TMDs. The putative IAA-binding sites and translocation surfaces of the plant ABCBs are relatively conserved, although they lack a high degree of sequence identity (Bailly et al., 2012).

Members of PIN-LIKES (PILS) family transporters were shown to function on endomembrane [mainly endoplasmic reticulum (ER)] structures. Identification of this novel putative auxin carrier family was based on the predicted topology of PIN proteins (Barbez et al., 2012). Interestingly, PILS and PIN proteins share only 10–18% sequence identity and belong to distinct protein families. PILS proteins are presumably characterized by two hydrophobic transmembrane regions found at N- and C-termini. The two transmembrane regions flank a relatively short hydrophilic region (loop) with a presumable cytosolic orientation. The loop is less conserved and the most divergent part of the PILS sequences (Feraru et al., 2012). The involvement of PILS in PAT has not been fully clarified but members of this family (PILS6, PILS2, PILS3 and PILS5) have been shown to repress auxin signaling, restrict the nuclear availability and perception of auxin (Barbez et al., 2012). Recently, the ER-localized PILS6 has been show to gate nuclear auxin levels and perception in response to high temperature (Feraru et al., 2019).

The proteins mentioned above do not exhaust the list of membrane localized players that participate in auxin regulation. The tonoplast importer, WALLS ARE THIN 1 (WAT1), was shown to retrieve auxin from vacuoles, suggesting that they also might contribute to auxin homeostasis (Ranocha et al., 2013). In addition, the PM-localized NITRATE TRANSPORTER 1.1 (NRT1.1) is importing IAA during nitrate absence (Krouk et al., 2010).

Last but not least, in many instances, the developmentally crucial auxin maxima are generated *via* the directional auxin efflux facilitated by PINs, in particular, those localized asymmetrically on the PM. This distinct localization is achieved and regulated by vesicular trafficking machinery that mediates directional exocytosis (Łangowski et al., 2016), endocytic recycling, and vacuolar degradation (Kleine-Vehn et al., 2008; Zwiewka et al., 2011; Nodzyński et al., 2013) of those carriers. Vesicles carrying PIN proteins and other PM cargos are formed on donor membranes by the coordinated action of several groups of proteins: small GTPases of ARF/SAR1 family, their guanine-nucleotide exchange factors (ARF-GEFs), GTPase-activating proteins (ARF-GAPs) and coat proteins (Singh and Jürgens, 2018). Among ARF-GEFs, GNOM is often reported as a general regulator of protein secretion since a fungal toxin Brefeldin A (BFA) impairs GNOM function and leads to the formation of intracellular structures composed of aggregated early secretory compartments containing cargoes (Lam et al., 2009), which would normally be recycled back to the PM by exocytosis (Geldner et al., 2003). PINs also aggregate after BFA treatment. However, it is worth highlighting that GNOM plays a role in PIN recycling to the basal but not the apical side of cells (Kleine-Vehn et al., 2008).

The importance of subcellular dynamics facilitating PIN polar localization on the PM with its downstream connection to establishment of auxin accumulation patterns guiding development has been abundantly reported (Nodzyński et al., 2012; Luschnig and Vert, 2014; Naramoto, 2017). Therefore, in this review, we would like to focus on the structure-function connections in PINs that are still insufficiently characterized, primarily discussing amino acid motifs from *Arabidopsis* carriers for which most extensive work has been done.

## PINs are Membrane Proteins with Two Helical Regions Linked by a Loop

IAA, as well as its synthetic analogs, are weak acids and therefore dissociate in relation to the pH. Based on this characteristic, a chemiosmotic polar diffusion model was proposed (Goldsmith, 1977) postulating that IAA in the low pH of the apoplast (approx. 5.5) is undissociated (IAAH) and able to permeate the PM without any transporters. However, once the molecules have passively diffused into the higher pH (approx. 7.0) of the cytoplasm, they dissociate forming IAA anions that cannot escape the cell unless *via* protein efflux carriers.

It was discovered that PIN proteins fit very well to the chemiosmotic hypothesis since some of them are asymmetrically localized in cells (Gälweiler et al., 1998; Friml et al., 2002b). Later, the capacity of PINs to transport auxin was tested in *Arabidopsis* and also in different heterologous systems (Petrášek et al., 2006; Yang and Murphy, 2009; Zourelidou et al., 2014). The developmental significance of auxin transport is reflected in *Arabidopsis pin1* loss-of-function mutants, which fail to develop floral organs properly and instead generate characteristic naked, pin-like inflorescences, which gave the name PIN-FORMED (often abbreviated as PIN) to this entire protein family (Okada et al., 1991; Gälweiler et al., 1998). In *Arabidopsis*, eight PINs have been identified and they can be divided into two groups, PIN1, 2, 3, 4, and 7, which localize asymmetrically at the plasma membrane of cells (Adamowski and Friml, 2015), and the remaining PIN5, PIN6, and PIN8 that also show localization on the endoplasmic reticulum membrane. At the ER, they mediate auxin exchange between the cytosol and the ER lumen (Barbez and Kleine-Vehn, 2013).

**Figure 1:**
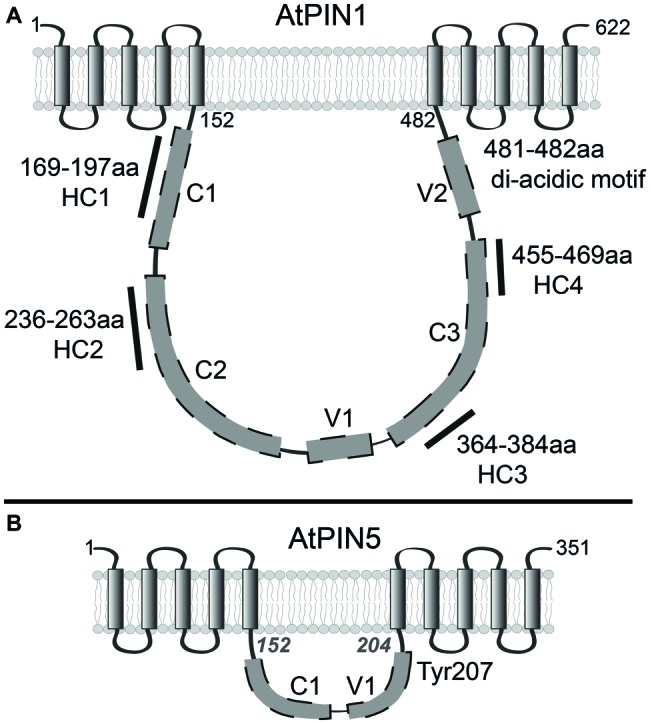
Schematic representation of *Arabidopsis thaliana* long and short PINs. **(A)** Topology of PIN1. Parts of hydrophilic loop marked with gray boxes indicate approximate arrangement of predicted conserved – C and variable – V regions. The positions of highly conserved, canonical motifs HC1–HC4 are indicated with solid lines and amino acid range indications. **(B)** Experimentally not verified topology model of PIN5 with 10 transmembrane domains separated by short hydrophilic region. Amino acid positions in italics indicate the predicted beginning and end of short HL. The tyrosine (Tyr) aligning within the putative tyrosine motif is indicated at position 207.

PINs are integral membrane proteins and it has been experimentally verified that the *Arabidopsis* PINs 1–4 have 10 TMDs grouped in two regions of five alpha helices separated with a large hydrophilic loop (HL) localized in the cytoplasm (Figure 1A; Nodzyński et al., 2016). Most of the experiments in those topology studies utilized PIN1 due to the availability of three versions of the protein with GFP inserted in different positions of the HL (GFP-1, 2, and 3). Fluorescence of those reporters was not affected by lowering of the apoplastic pH indicating their cytoplasmic localization along with the HL into which they were inserted. Those results were corroborated by immunolocalization of the HL and the C-terminus in membrane permeable and non-permeable conditions. When PM permeability for the antibodies was reduced, the HA tagged C-terminus was preferentially decorated, while HL showed significantly lower labelling, confirming the cytoplasmic versus apoplastic localization of the HL and C-terminus respectively. Those empirical data limited the amount of valid topological predictions, supporting a 10-TMD PIN topological model. However, the exact position or length of each TMD was not investigated in detail. To gain this information more constructs with tag insertions in the minor loops, linking the helices, would need to be tested. It is unlikely, but not impossible that the total number of TMD may not be 10, just as long as particular PIN parts are positioned accordingly with the experimental data. One such case was published by Wang et al. (2014) depicting a 9-TMD PIN model with the HL and C-terminus positioned on opposite sides of the PM. Such topology was possible when one TMD from the N-terminal helical region was omitted. Not most, but indeed several topology prediction programs indicate such scenario although, usually they depict the N-terminus and the HL as both facing outside the cell which does not correspond with experiments. Also, the topology prediction reliability of the TMDs 1–5 was above 90% (Nodzyński et al., 2016). Still, experimental verification of the N-terminuses position was not done. We were unable to perform it since in our hands N-terminal GFP tagging of PINs was not yielding functional proteins, presumably due to disruption of the PM retention signal. It is also worth to mention that, in the topology studies (Nodzyński et al., 2016), the transmembrane regions were, rather casually, called “bundles” in the sense that there are two groups of five helices. The term “bundles” was not aiming to make deeper structural inferences for which more detailed studies would be required, if one would like to assess which alpha helices bundle together or interact with each other. The same can be stated for the ER-localized carriers for which the topological predictions are even more variable, and their topology has not been verified experimentally thus far. The PIN5 and PIN8 harbor a relatively short stretch of hydrophilic amino acids between their transmembrane domains (Figure 1A; Viaene et al., 2013). Notably, the hydrophilic loop of PIN6 is of intermediate length in comparison to the so-called “long-loop” PINs (PIN1–4 and PIN7) vs. the ER “short-loop” PINs and exhibits a dual ER and PM localization (Simon et al., 2016). The predicted structure of the “long” PINs is similar to the structures of secondary transporters that use an electrochemical gradient across the membrane to facilitate transport (Křeček et al., 2009) sharing also a limited sequence similarity with some prokaryotic (Kerr and Bennett, 2007) and eukaryotic transporters (Gälweiler et al., 1998; Palme and Gälweiler, 1999). Consequently, no ATP binding cassettes have been identified in PIN proteins. There is also experimental data implicating the proton gradient across as the PM as important for auxin transport (Rubery and Sheldrake, 1974; Hohm et al., 2013). However, there are no biochemical studies unequivocally clarifying that the H^+^ are the driving force for PIN-mediated auxin efflux.

## PIN Transmembrane Domains Show High Sequence Conservation

Transmembrane domain regions of PINs in *Arabidopsis,* as well as in *Plantae,* are much more conserved than the hydrophilic loop linking them (Křeček et al., 2009; Bennett et al., 2014). Alignment of *Arabidopsis* PINs reveals greater conservation of amino acids in the first (N-terminal) alpha helical region when ER-localized PIN5 and 8 are aligned to the PM-localized PINs 1–4 and 7. However, both the PM PINs (Petrášek et al., 2006) and the ER ones (Mravec et al., 2009; Ding et al., 2012) seem to have the capacity to transport auxin, suggesting that the auxin translocation activity is encoded in the transmembrane domains and not in the hydrophilic loop, which is prominently shortened for the ER PINs. Notably, the transport assays for ER-localized PINs were based on auxin metabolic profiling, showing increased conjugation of IAA in case of PIN5 over-expression (Mravec et al., 2009) while the converse was reported when PIN8 was over-expressed (Ding et al., 2012). Activity assessment of membrane proteins requires their incorporation in a lipid bilayer across which transport can commence. Establishment of such setup is more challenging in comparison to an activity assay of soluble enzymes. However, it would be interesting to reconstitute the ER PINs in liposomes (Nimigean, 2006) to test their activity. Such, more biochemically pure setup would further support the conjecture that PINs are independent efflux carriers and enable to evaluate more precisely their transport specificity.

The length of the TMDs was also analyzed by aligning so-called “canonical” PIN sequences from multiple plant species. Thus, the predicted length of the N-terminal TMD region was very consistent, ranging from 155 to 176 amino acids. The longer sequences, originating mostly from the *Poaceae* family, were usually extended by insertion of up to 18 amino acids between positions 97 and 98 falling in between third and fourth alpha helix of the generalized PIN model. Similarly, predicted C-terminal TMDs were even more consistent with the length of 154 amino acids (Bennett et al., 2014). Calculated frequency of the most common amino acids at each core position in the TMDs, in all canonical PINs, indicated 106 invariant or near-invariant (>99% amino acid identity) positions, and 87 amino acids with more than 90% identity. The predicted TMDs positions did vary but they were still the most highly conserved parts of the whole transmembrane domain region with only helix 3 showing less conservation in the generalized PIN model. The conservation of TMDs likely was evolutionarily selected in relation to the stabilization of the protein within the lipid bilayer or its auxin translocation activity (Bennett et al., 2014).

**Figure 2:**
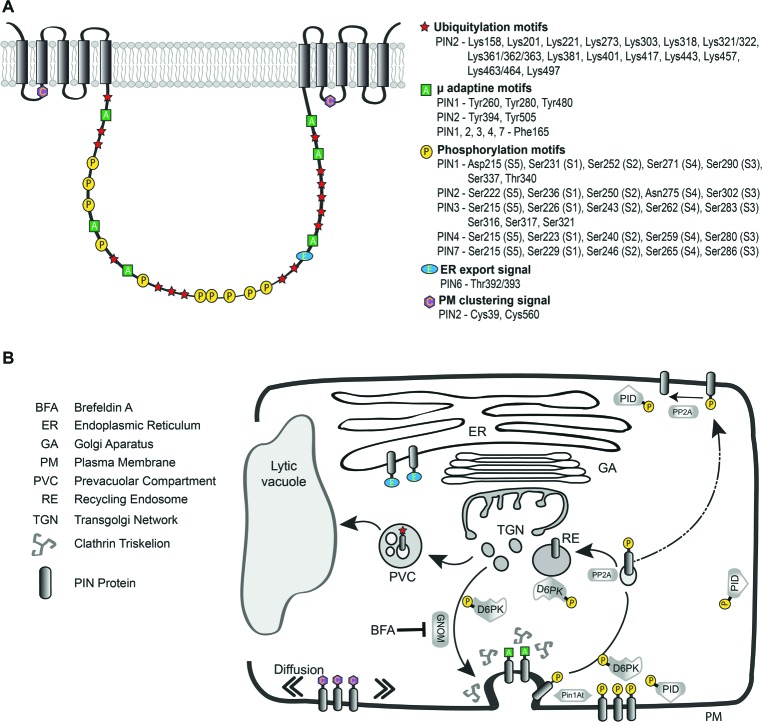
Schematic designation of known sequences and residues in a generalized model of *Arabidopsis thaliana* long PINs and their role during intracellular trafficking. **(A)** Symbols indicate the functional elements and their approximate location in the canonical PIN model, precise positions of those motifs for particular PINs are listed on the right. **(B)** Simplified illustration of PIN vesicular trafficking with the functional motifs indicated at those steps of the intracellular pathway in which they play most prominent roles. The ER export signal present in the long hydrophilic loop of the PIN protein is oriented into the cytoplasm and serves as a signal for further secretion. GNOM is a regulator of protein recycling from early endosomal compartments like TGN and its inhibition by BFA impairs exocytosis. The μ-adaptin motifs in the HL participate in interaction with clathrin machinery. PIN phosphorylation status controlled by kinases (PID, D6PK) and phosphatase (PP2A) is crucial for cycling, PM localization and activity of the auxin efflux carrier. Pin1At-facilitated isomerization of proline residues in the vicinity of phosphorylation sites affects PIN1 polar localization. Cysteines present in small cytosolic loops linking the helices play a role in PIN PM diffusion and trafficking. Ubiquitylation serves as a signal for vacuolar degradation of the carrier.

It is worth noting that the eight very small loops linking the alpha helices, while being more variable, still contain several highly conserved residues which might play important structural or regulatory roles. In congruence with this assumption, recently two evolutionarily-conserved cysteine residues (C39 and C560; Figure 2A) have been implicated in regulating PIN2 endocytosis and distribution on the PM. PIN2 mutant version with cysteine-to-alanine mutations shows more cytoplasmic fluorescent signals and more variability in abundance of the efflux carrier on the PM. The mutation also caused more wavy root while the overall gravitropism was not altered. A detailed microscopic analysis of *pin2^C39,560A^:Venus* double mutant revealed modified PIN2 distribution within plasma membrane microdomains (clusters), indicating overall changes in mobility of the protein (Retzer et al., 2017). Indeed in comparison with different apolar cargoes, the lower PM diffusion of PINs, together with their targeted super-polar exocytosis and the endocytosis collecting laterally diffusing auxin carriers, have been proposed as mechanisms contributing to PIN polarity maintenance (Figure 2B; Kleine-Vehn et al., 2011). In addition, it has been shown that PIN5 fused with PIN2-HL is able to localize ectopically on the PM, but does not exhibit PIN2-like, polar localization (Ganguly et al., 2014). All those reports seem to indicate that functional elements could be encoded in the small loops linking the alpha helices or in the TMDs themselves, together participating in PIN polarity establishment. This process might also involve interactions between PINs and the cell wall, as protoplasting of epidermal cells has resulted in PIN2 polarity disappearance (Feraru et al., 2011). Therefore, more detailed studies of PIN structure would be instructive in finding out, what motifs play a role in PIN polarity maintenance.

## The Hydrophilic Loop Sequence is Less Conserved Yet with Identifiable Motifs

Based on the size of the central hydrophilic loop, PINs can be tentatively divided into two major subgroups, the “long” and “short” ones (Viaene et al., 2013). The HL sequence is much more divergent than the TMD regions (Křeček et al., 2009), but already one of the initial multi-species alignments revealed conserved domains (C) and variable regions (V) in the HL (Figure 1A; Zažímalová et al., 2007). The authors postulated that long HL includes conserved domains C1, C2, and C3 and variable regions V1 and V2 depicting their schematic, approximate distribution within the HL and already noting that the motifs appear in a particular order. In this report, the short HL was characterized by the presence of only the domain C1 and region V1 (Figure 1B). The variable regions were homologous within the group of long HL PINs but, they differed significantly when compared between short and long looped PINs.

Latter more detailed studies were published. Wang et al. (2014) identified 20 motifs after aligning PIN1 proteins sequence from multiple species. The two longest motifs (M) were aligning mostly in the TMD regions, M1 in the N-terminal and M2 in the C-terminal part of the PIN1 protein, again indicating strong conservation of the helices across species. Several shorter motifs were identified in the HL showing that also this region has cross-species conservation. Notably, some of the shorter conserved sequences (M4, M13, M9) encompassed the phosphorylation sites S1–S4 (Barbosa et al., 2018) present in the *Arabidopsis* PM PINs as well as the S337, T340 residues (M11) shown to control polar sorting of PIN1 (Figure 2A; Zhang et al., 2010). In the same year, another study focused on the HL region of PINs in multiple plant species. This alignment of PIN sequences has identified four highly conserved (HC) motifs HC1–HC4 in the central HL (Figures 2A,B; Bennett et al., 2014). Authors reported that long loop usually harbors all four conserved motifs, always appearing in succession. Notably, the HC4 region was also partially present in the short HL of PIN5 and PIN8 within angiosperm kingdom. Authors of this study designated particular PIN proteins as “canonical” if they matched the consensus sequence of all four HC motifs with at least 50% identity or 70% similarity. Auxin efflux carriers not fulfilling these criteria were named “noncanonical” (Bennett et al., 2014). With this classification, authors strove to describe more accurately the structural features of PIN proteins, not referring only to the length of the HL, but rather to the content of its sequence. Still, most of the canonical PINs did have long HLs. Using this nomenclature PIN5 and 8 would be noncanonical, while PIN6 might be classified as “semi-canonical”.

Authors also stipulated that noncanonical PINs evolved repeatedly by sequence divergence from canonical precursors. They pointed out the unique structural features of *Arabidopsis* PIN5 and PIN8 indicating that they cannot be classified as ancestral precursors of canonical PINs, as surmised prior (Viaene et al., 2013). Indeed, the *Arabidopsis* ER-localized PINs have a more divergent composition of the TMD than the PM-localized ones. Also, in case of *Arabidopsis* PIN5 and PIN8 the topology predictions are much more variable than for PM PINs 1–4 and 7. As mentioned above, a chimeric version of PIN5 harboring the HL of PIN2 was able to more efficiently reach the PM, but without exhibiting the characteristic polar localization (Ganguly et al., 2014) hinting at the functional distinction of ER PINs from the PM ones, supporting their divergent evolution.

## The Hydrophilic Loop Harbors Regulatory Cues for PIN Trafficking and Activity

Numerous studies indicated the presence of information necessary for PIN trafficking and polar targeting within the HL amino acid sequence (Figure 2B; Michniewicz et al., 2007; Huang et al., 2010; Zhang et al., 2010; Ganguly et al., 2014). Congruently, the short looped PIN5 and 8 show predominantly ER localization, thus it is presumed, that they lack the molecular cues for PM trafficking (Ganguly et al., 2014). The experimentally verified cytosolic orientation of the HL is also sensible from the functional point of view as it makes the loop accessible for the cytoplasmic, subcellular trafficking machinery. Motifs in the HL are involved in clathrin-mediated endocytosis (Kleine-Vehn et al., 2011; Nodzyński et al., 2016; Sancho-Andrés et al., 2016) and being a target of phosphorylation by cytosolic kinases (Dhonukshe et al., 2010; Huang et al., 2010) as well as ubiquitylation (Abas et al., 2006; Leitner et al., 2012a), which collectively modulate the intracellular trafficking, PM stability and polar delivery of long *Arabidopsis* PINs. Those processes contribute to the regulation of PIN-mediated directional auxin translocation, which can take place when those efflux carriers are present at the PM, distributed in an asymmetrical fashion and active (Figure 2B; Wiśniewska et al., 2006; Zourelidou et al., 2014). Below we will discuss some of the amino acid motifs contained in the HLs and their roles uncovered so far.

### Tyrosine Sorting Motif Is Involved in PIN Trafficking

Plasma membrane resident PIN proteins undergo clathrin-dependent endocytosis (Dhonukshe et al., 2007) regulating their PM abundance (Adamowski et al., 2018) and maintaining their polar localization (Kitakura et al., 2011; Kleine-Vehn et al., 2011). Cargo sorting into clathrin-coated vesicles is mediated by adaptor protein (AP) complexes, which recognize cytosolic sorting signals in membrane proteins *via* their medium (μ) subunits (Robinson, 2015). The *Arabidopsis* genome encodes adaptin subunits of 4 types of putative AP complexes (AP-1 to AP-4) including five medium subunits, named μA (μ2), μB1 (μ1–1), μB2 (μ1–2), μC (μ4), and μD (μ3) (Boehm et al., 2001; Park et al., 2013). One of the best- characterized sorting signals, which is recognized by the medium (μ) subunit of adaptor complexes, is the YxxΦ motif, where Y denotes the single letter abbreviation for tyrosine, “x” is any amino acid, and Φ is a bulky hydrophobic residue leucine, isoleucine, phenylalanine, methionine or valine (Canagarajah et al., 2013). In a different type of tyrosine-based motif (NPxxY) (Traub, 2009), Tyrosine 480 (Y-480) was proposed to be important for PIN1 localization, since mutagenesis of the amino acid stretch containing this tyrosine (NPNSY to NSLSL), caused PIN1 retention at the ER membrane (Mravec et al., 2009). This tyrosine residue and the motif itself is found in all long HL PINs and seems to be important for PIN1 trafficking and localization (Sancho-Andrés et al., 2016). Interestingly, similar tyrosine containing conserved region was also identified in the short hydrophilic loop of PIN5 and PIN8 (Figure 1A; Mravec et al., 2009).

Similarly, the clathrin-dependent PIN internalization was selectively affected by mutating a conserved tyrosine residue of PIN2 required for cargo-specific sorting into clathrin-coated pits (Figures 2A,B). While the majority of *PIN2^Y505A^-YFP* was localized to the apical cell side in root epidermal cells, the mutation strongly enhanced PIN2 lateral localization. Notably, *pPIN2:PIN2^Y505A^-YFP* showed reduced PIN internalization and did not fully rescue the *pin2* mutant phenotype (Kleine-Vehn et al., 2011).

Further search for putative sorting signals in HL of PIN1, identified a phenylalanine (F)165 and three tyrosines in positions 260, 328, and 394 participating in the interaction with several μ-adaptins, thus possibly being involved in PIN1 trafficking and polar localization (Figures 2A,B). However, only F165, present in all canonical PINs, which has the ability to interact with μA- and μD-adaptins *in vitro*, appeared to be essential for the routing and localization of the efflux carrier *in vivo*, since the PIN1:GFP-F165 mutant showed reduced endocytosis, but also accumulated in intracellular structures (Sancho-Andrés et al., 2016). Nevertheless, PIN1:GFP-F165A mutant was still internalized, therefore other residues in the HL may also participate in PIN1 endocytosis. Even though, precise function of AP-3 in PIN1 trafficking needs further research, the PIN1-GFP accumulation in big intracellular structures present in a μ3-adaptin mutant (Sancho-Andrés et al., 2016) and in mutants of other subunits of the AP-3 complex (Feraru et al., 2010; Zwiewka et al., 2011), solidify the role this complex in PIN1 trafficking and localization.

### ER Exit

Membrane-localized proteins are synthesized on the rough ER and remain integrated into the lipid vesicles as they traffic to their final destinations along the secretory pathway. A starting point of unraveling which protein motifs retain PINs at the ER membrane and which are necessary for ER exit, was provided during the investigation of PIN5 localization. Alignment of short-ER PINs and long-PM ones revealed the presence of a conserved amino acid stretch, named the di-acidic motif presumably, important for trafficking of proteins from the ER (Mravec et al., 2009). In PIN1 HL this sequence is abstracted as NPxxYxxΦ, where “x” represents any amino acid and Φ denotes a residue with a bulky hydrophobic side chain, the other letters designate specific amino acids. The di-acidic motif is composed from the tyrosine-480 motif NPNSY followed by SSL sequence (Figure 2A). Alignment of the region spanning the tyrosine motif of all *Arabidopsis* PIN proteins revealed a conserved sequence NPN(S/T)YSSL (where S is found in PIN1 sequence and T is present in other long PINs) in the HL of canonical PINs, but the last three SSL amino acids are missing in the short-looped PIN5 and PIN8. The attempt to mutagenize the di-acidic motif in PIN1 resulted in significant accumulation of the protein at the endoplasmic reticulum (Mravec et al., 2009), supporting its role in ER exit. It is important to highlight however, that the mutagenesis targeted also the conserved tyrosine residue (NPNSY mutated to NSLSL) which could interfere with clathrin mediated endocytosis in general (Ohno et al., 1995; Bouley et al., 2003). On the other hand, manipulations of PIN1 HL residues, especially the F-165 mutation, interacting with the μ-adaptin endocytic machinery resulted in accumulation of the carrier in round, condensed structures (Sancho-Andrés et al., 2016). Whereas, in PIN1-GFP-tyr (NSLSL) line, PIN protein was clearly localized at the ER (Mravec et al., 2009). Nevertheless, it cannot be ruled out that mutagenesis of this specific sequence would result in improper folding and protein retention at the ER.

Interestingly, phosphorylation-dependent ER exit has been recently reported for PIN6 protein. Functional analysis of threonine residues (T) 392 and 393 (Figure 2A; Benschop et al., 2007), phosphorylated by mitogen-activated protein kinase (MAPK) MPK4 and MPK6 *in vitro* (although T393 is not phosphorylated by these kinases) revealed a key role of indicated residues in PIN6 ER exit. Those phosphorylation sites also play a role in main root and root hair growth regulation, as well as development of the inflorescence stem at the appropriate, possibly environmentally determined, time (Ditengou et al., 2018). This demonstrates yet another facet of phosphorylation controlling PIN subcellular trafficking beside the earlier discovered control of their PM polar localization.

## PIN Post-Translational Modifications

Post-translational modifications (PTM) play an important role in regulating protein folding, activity by modifying the targeting to specific subcellular compartments, and interaction with ligands or other proteins (Spoel, 2018). The early steps of processing and sorting of *de novo* synthesized PIN proteins are still largely unknown. PIN post-translational modifications such as glycosylation, an indicator of protein maturation, or proteolytic processing, including signal peptide recognition and cleavage, have not been extensively experimentally verified (Luschnig and Vert, 2014). It is worth noting that online bioinformatic tools and databases can be instructive in search for known and potential sites of post-translational protein modification. Some of recently launched user-friendly bioinformatics online resources: iPTMnet (Huang et al., 2018) and Plant PTM Viewer (Willems et al., 2018) integrate PTM information from text mining, curated databases and ontologies providing visualization tools for exploring PTM networks, conservation across species or even crosstalk. One of the most common PTMs is protein phosphorylation. The database of Phopsho-sites in PlanTs (dbPPT) (Cheng et al., 2014), containing experimentally identified sites in plant proteins, indicates 77.99, 17.81, and 4.20% of phosphorylated serine, threonine and tyrosine residues respectively. Consequently, also for PINs, many phosphorylation sites have been discovered and will be also discussed below.

### Extensive PIN Glycosylation Has Not Been Reported

An early review of multiple PIN sequences with the Prosite database (Hulo et al., 2006) suggested two clusters of sequences in the HL region containing potential glycosylation motifs (Zažímalová et al., 2007). They are however positioned in the HL region, which we now know, is in the cytosol (Nodzyński et al., 2016), and it is more likely that parts of the protein facing the lumen of ER and Golgi would be glycosylated and not the cytoplasmic ones. Congruently, the theoretical size of PIN2 (~70 kDa) matched with the observed band on the SDS PAGE gel, indicating no extensive glycosylation of the protein (Müller et al., 1998).

### Several Residues in the Hydrophilic Loop of PINs Are Phosphorylated

Protein phosphorylation is an enzymatic reaction involving the addition of a phosphate group (-PO_4_)^3−^ to the polar residue -R of various amino acids (usually serine, threonine, and tyrosine, or histidine). This covalent modification is often associated with protein activity regulation related to its conformational change, allowing to interact with other molecules, proteins, and even assemble or uncouple protein complexes (Sacco et al., 2012; Ardito et al., 2017). Kinases (phosphotransferases) reversibly attach a phosphate group while phosphatases do the opposite by hydrolysis.

Three Ser/Thr protein kinase families were reported to be involved in phosphorylation of PINs: AGC kinases (serine/threonine kinases homologous to mammalian protein kinase A, cGMP-dependent kinase, and protein kinase C) (Bögre et al., 2003) belonging to the AGCVIII subfamily (Galván-Ampudia and Offringa, 2007), MITOGEN-ACTIVATED PROTEIN (MAP) KINASES (MPKs/MAPKs) (Jia et al., 2016; Dory et al., 2017), and Ca^2+^/calmodulin-dependent protein kinase-(CDPK)-related kinases (CRKs) (Rigó et al., 2013). Among the AGCVIII subfamily, two subgroups have been directly implicated in PIN-mediated auxin transport: PINOID (PID) together with its presumed functional paralogs WAG1, WAG2 (named after *wag* mutants that showed root waving) (Santner and Watson, 2006) as well as D6 PROTEIN KINASE (D6PK) with the three candidate paralogs D6PK-LIKE (D6PKL) 1–3 (reviewed in Willige and Chory, 2015).

Like *pin1* plants (Okada et al., 1991; Gälweiler et al., 1998), PID mutants exhibit the pin-shaped inflorescence (Bennett et al., 1995; Christensen et al., 2000; Benjamins et al., 2001). PID and the closely related WAGs also play roles in hypocotyl phototropism (Ding et al., 2011; Haga et al., 2014), gravitropism (Grones et al., 2018) as well as root gravitropism (Ganguly et al., 2012) and, apical hook opening (Willige et al., 2012). In all those processes PIN polarity has to be modified to redirect auxin flux, facilitating differential growth. The polarity related action of AGCVIII kinases is in *Arabidopsis* counteracted by the protein phosphatase 2A (PP2A) complex, and defects in the regulatory subunits - PP2AA1, PP2AA2, and PP2AA3 result in similar phenotypes as the PID overexpression (Garbers et al., 1996; Michniewicz et al., 2007; Xi et al., 2016). Within the *Arabidopsis* AGCVIII subfamily, the D6PK and related D6PKL1–D6PKL3 (D6PKs) are also required for auxin transport-dependent processes, such as hypocotyl phototropism, negative gravitropism, shade avoidance as well as lateral root and shoot differentiation (Zourelidou et al., 2009; Willige et al., 2013; Kohnen et al., 2016). Interestingly, their phosphorylation function affects PIN-mediated auxin efflux activity, rather than the PM polarity of those carriers (Zourelidou et al., 2009, 2014).

It has been reported that PID action can be regulated by interaction with different Ca^2+^ binding proteins (Benjamins et al., 2003) suggesting a possible role of Ca^2+^ signals in PIN polarization. What is more, mutations or chemical treatments which elevated Ca^2+^ levels, were associated with PID-related polarity shifts of PIN1 in the stele and PIN2 in the cortex cells, but not in the epidermis (Zhang et al., 2011). Consequently, Ca^2+^ was implicated in endomembrane trafficking in plants (Himschoot et al., 2017), but how those signals translate into PIN polarity is not fully understood. In the case of PIN2, the calcium-dependent CRK5 may be a part of the machinery translating Ca^2+^ levels into PIN polar targeting (Rigó et al., 2013). *In vitro* experiments confirmed PIN2 phosphorylation by CRK5, but the respective phosphosites have not been mapped. In *crk5* mutants, PIN2 protein was partially depleted from the apical plasma membrane in epidermis cells and showed polarity defects in the cortex displaying apolar or outer-lateral PIN2 localization, but for other PINs the effect was not observed (Rigó et al., 2013). Upon treatment of the *crk5* mutants with BFA, which is blocking exocytosis of PM cargos, PIN2 showed accelerated accumulation in BFA compartments (Rigó et al., 2013). Moreover, in *crk5*, inhibition of primary root elongation and delay of gravitropic bending of root and shoot were observed. Those data are suggesting that the CRK5 function is ether to inhibit PIN2 endocytosis or to activate its recycling (Rigó et al., 2013).

### PIN Phosphorylation as the Hypothetical Down-to-Up-Polarity Switch

The most evident phenotypical aberrations are those related to PIN1 function and the phosphorylation sites in the HL of this carrier are probably most comprehensively experimentally verified. The kinases PID/WAGs and PP2A phosphatase were shown to regulate the phospho-status of the evolutionary conserved S1-S3 residues in PIN1 HL (TPRxS where “x” is S or N, see Figure 2A). Phosphorylation of those sites resulted in PIN apicalization (shootward localization) on the PM of cells while de-phosphorylation caused a predominantly basal (rootward) localization of the carrier (Figure 2B; Friml et al., 2004; Michniewicz et al., 2007; Kleine-Vehn and Friml, 2008). In connection, it has been observed that in *pid* mutants, PIN1 does not relocalize to the apical plasma membrane to redirect auxin distribution during shoot differentiation (Friml et al., 2004). Consequently, S1–S3 phosphosite mutations interfere with the ability of PIN1 to rescue *pin1* inflorescence phenotype (Dhonukshe et al., 2010). In the wild type root epidermal cells, PIN2 is apical, but in *pid*, *wag1*, *wag2* mutants it is basally localized (Dhonukshe et al., 2010). What is more, in *Arabidopsis* root, PID/WAG overexpression results in a basal to apical PIN polarity shift, which correlates with root meristem collapse due to loss of the root tip auxin maximum (Michniewicz et al., 2007; Dhonukshe, 2011; Weller et al., 2017). S1–S3 phosphosite mutations abrogate the effects of PID overexpression on PIN1 polarity, and phosphorylation-mimicking mutations result in constitutive apicalization of the PIN protein (Dhonukshe et al., 2010; Huang et al., 2010). Importantly, phosphomimetic mutations of PIN3 HL in the previously described conserved TPRxS motif resulted in defective root and hypocotyl gravitropic growth as well as inhibition of gravity-induced PIN3 relocation similar to the effects observed in PID overexpression lines (Grones et al., 2018). Based on the results described above, mostly concerning PIN1, a quite long-standing hypothesis of a binary polarity switch mechanism was proposed in which more phosphorylation resulted in apical PIN trafficking while less phosphorylation made the carrier remain on the basal cell site. However, during those studies there were no tools available to probe PIN phospho-status directly at a given subcellular localization. This knowledge gap was filled more recently and is discussed below.

### D6PKs and Phosphosite Antibodies – Updating the PIN Polarity Model

Similarly to PID/WAGs, D6PK phosphorylates PIN proteins at serines S1–S3 as well as at two additional serine residues, S4 and S5 (Figure 2A; Zourelidou et al., 2009, 2014; Willige et al., 2013). The S4 and S5 are conserved in PIN3, PIN4, and PIN7, but S5 is absent from PIN1 while PIN2 lacks both S4 and S5 (Figure 2A; Zourelidou et al., 2014). Like PID, D6PK activates PIN-mediated auxin efflux (PIN1, PIN3), although with a slightly different PIN phosphosite preference, D6PK phosphorylating the S2 site less (Zourelidou et al., 2014). Unlike in the case of PID or WAG, D6PK overexpression does not result in PIN polarity changes that has been explained by different phosphosite preferences between D6PK and PID/WAGs (Zourelidou et al., 2009, 2014; Barbosa et al., 2014). Furthermore, recent investigations utilizing phosphosite-specific antibodies directed against PIN1 S1–S4 sites, immunodetected phosphorylated PIN1 at the basal as well as at the apical plasma membranes of root cells. The phosphorylation at the basal PM was strongly sensitive to BFA, rather indicative of D6PKs that is predominantly basally localized but not of the BFA insensitive PID/WAGs (Kleine-Vehn et al., 2009; Weller et al., 2017). Interestingly, when PIN1 was targeted to the apical plasma membrane using a GFP fusion impairing PIN1 trafficking (PIN1::PIN1:GFP-3), the efflux carrier was phosphorylated and the immunodetected P-signal was present even after BFA treatment (Weller et al., 2017). Those results are in agreement with the model of phosphorylation controlling PIN localization but it seems that the basal-to-apical polarity switch does not depend only on increased phosphorylation since apically localized PIN1-GFP-3 is also phosphorylated (Weller et al., 2017). Thus, phosphorylation might be only one of the steps that initiate PIN polarity shifts and it is conceivable that also other proteins participate in the sequence of events leading to PIN polar targeting. Since the HL constitutes almost half of the total size of PM PINs it may serve as a hub for multiple interactions that together with phosphorylation co-regulate PIN polar delivery also depending on the cellular context (Figures 2A,B; Wiśniewska et al., 2006).

### Pin1At Isomerase – Phosphorylation and Hydrophilic Loop Conformation Impacting PIN Polarity

Phosphorylation has been also linked to protein conformational changes (Humphrey et al., 2015). Interestingly, peptide bond *cis/trans* isomerization of prolines following phosphorylated serine or threonine (S or T–P) by Pin1At (Peptidyl-prolyl cis-trans isomerase NIMA-interacting), was connected to regulation of flowering time in *Arabidopsis* (Wang et al., 2010). The Pin1At accelerates the *cis/trans* conformational change of the phosphorylated Ser/Thr-Pro motifs in the central PIN1 hydrophilic loop and affects polar localization of PIN1 (Figure 2B). In other organisms, this isomerase rearranges only phospho-Serine/Threonine-Proline motifs and has been reported to bind to a subset of proteins, thus mediating a post phosphorylation regulatory function (Ping Lu et al., 1996; Abrahamsen et al., 2012; Brichkina et al., 2016).

In *Arabidopsis* Pin1At mediates the antagonistic effects of PID and phosphatases on PIN1 polarity. Overexpression of Pin1At enhances the agravitropic phenotype of *pp2aa1–6*, generating coiled roots, while downregulation of the isomerase in AmiR-Pin1At line suppresses root agravitropism of 35S:PID (Xi et al., 2016). Pin1At most effectively isomerizes the S337, found in the last motif in PIN1-HL out of 4 tested by Nuclear Magnetic Resonance (NMR) (Xi et al., 2016). This serine is targeted by the cascade of MAP Kinase activating the Mitogen-Activated Protein Kinase (MKK7–MPK) 3/6-targeted (Jia et al., 2016). Pin1At, to a lesser extent, also isomerizes the phosphorylated T227, T248, and T286, reported to be targets of MPK4 and MPK6 (Dory et al., 2017). Congruently with previously published data (Zhang et al., 2010), the downregulation of Pin1At suppresses the phosphomimetic mutations (serine to aspartic acid change) S337 and T340 in root stele cells, which are inducing a basal-to-apical shift of PIN1 (Xi et al., 2016). If this role is directly linked to PID remains to be shown because PID could not phosphorylate PIN1 S337 (Zhang et al., 2010). It is worth mentioning, the effects of the isomerase on the polarity of GFP tagged PIN2, 3, 7 were not observed (Xi et al., 2016). This leaves an open possibility for other mechanisms to control the polarity of different PINs. It is not unlikely that the regulatory elements are encoded in the sequence context, extending beyond the phosphorylation sites and into the structural elements of HLs that have not been proven or experimentally investigated yet.

### PIN Ubiquitylation

After internalization from cell surface, proteins can either be recycled back from Trans-Golgi Network (TGN) to PM, or sorted to Multivesicular bodies (MVB) and further routed towards the vacuolar proteolysis (Figure 2B). In plant cells, the ubiquitylation status seems to signal which membrane cargo will traffic back to PM and which will be degraded (Tian and Xie, 2013). A single molecule of ubiquitin (Ub), or a poly-Ub string, can be attached to a lysine side chain of the targeted protein *via* a C-terminal carboxylate group of ubiquitin (Ub). As mentioned, the Ubs can also be chained together *via* an isopeptide bond linking one of seven lysines (K) of the already bound Ub with the C-terminal glycine of second Ub molecule. Several lysine residues of the cargo protein can be ubiquitinated. The K63-linked ubiquitin chains are generally considered to be critical signals for endosomal-mediated sorting and vacuolar degradation of PM proteins (Reyes et al., 2011; Isono and Kalinowska, 2017).

One of the first immunoprecipitation experiments demonstrated ubiquitylation of a considerable range of PM proteins, including PIN2, but no clear link to PIN protein fate has been established at that point (Abas et al., 2006). Thus far, only one report systematically investigated possible lysine residues targeted by K63-linked polyubiquitylation in PIN2 protein (Leitner et al., 2012a). In this study, authors prepared a collection of PIN2 constructs having increasing numbers of mutated lysines and tested their capacity to rescue the agravitropic *pin2*/*eir1–4 (pin-formed/ethylene insensitive root 1)* mutant phenotype. Importantly, it was shown that single K mutations and combinations of only a few lysine-to-arginine (R) exchanges did not visibly interfere with PIN degradation. However, combining multiple K-to-R point mutations in the hydrophilic loop did elevate the PM stability of the modified PIN2 to the point that it failed to fully complement the agravitropic *eir1–4* mutant. Consistently, the more lysines in the loop were replaced, the more apparent this effect became. Two constructs that were least effective in rescuing the *eir1–4* had 12 and 17 lysines mutated in amino acid positions (*pin2*^12K-R^) *158, 201*, *318*, *321*, *322*, *361*, *362*, *363*, *381*, *497*, *556*, *614* and (*pin2*^17K-R^) *221*, *273*, *303*, *318*, *321*, *322*, *361*, *362*, *363*, *381*, *401*, *417*, *429*, *443*, *457*, *463*, *464* (Figure 2A). Overall, these findings imply that K63-linked ubiquitin chain modification, of multiple lysines situated in the hydrophilic loop, redundantly control PIN2 degradation and root gravitropism (Leitner et al., 2012a). This is consistent with the previously reported vacuolar trafficking of PIN2 in epidermal cells of the convex side of the root during root gravitropic bending, decreasing auxin flux and causing elongation of those cells (Kleine-Vehn et al., 2008). Interestingly, five of above-mentioned lysines (*PIN2: 158, 201*, *363*, *497*, *614*) are conserved among *Arabidopsis* PIN1–4 and 7, hence could be investigated in other PINs.

## PIN – ABCB Interactions

Besides PINs, ABC transporter B subfamily proteins (ABCBs, previously named PGPs - p-glycoprotein subfamily) also participate in auxin efflux from plant cells. ABCBs are primary transporters using adenosine triphosphate (ATP) to power auxin translocation. What is more, physical interactions of those auxin carriers were demonstrated by Y2H and co-immunoprecipitation analyses using C-terminal domains of ABCB1 and ABCB19 pairing with the hydrophilic loops of PIN1 and PIN2, respectively (Blakeslee et al., 2007; Rojas-Pierce et al., 2007). Although PINs are able to efflux auxin on their own (Petrášek et al., 2006), when co-expressed as PIN1-ABCB1 and PIN1-ABCB19 pairs, elevated export rate and increased substrate specificity was reported for PIN1 in comparison with the single transporter assays. While, co-expression of ABCB1 and 19 enhanced only the substrate specificity of PIN2 (Blakeslee et al., 2007), expressing ABCB4 with PINs had an influence on transport directionality. However, the interaction has not been proven biochemically thus far (Bandyopadhyay et al., 2007; Blakeslee et al., 2007). Additional evidence for PIN and ABCB functional interactions came from microscopy-based cell biological studies showing partial co-localization of ABCB1 with PIN1 in the stele and with PIN2 in cortical and epidermal cells of the *Arabidopsis* seedling roots (Blakeslee et al., 2007). Also, genetic analyses were conducted, utilizing mutant combinations, to confirm the ABCB-PIN interactions however, those efforts unraveled yet another layer of complexity (Geisler et al., 2017). Shoot and leaf defects observed in *abcb19 pin1* double mutant were stronger than in *pin1* alone. Notably, they were partially restored in the triple *pin1 abcb1 abcb19* mutant, presumably as a result of ectopic auxin accumulation in the shoot apical meristem (Blakeslee et al., 2007). The *pin2 abcb1 abcb19* triple mutant showed enhanced gravitropism defects in comparison with *pin2* single. Synergistic action could be observed in *pin1 abcb1 abcb19* triple mutants cotyledon patterning showing more severe defects than *pin1* single or *abcb1 abcb19* double combinations (Mravec et al., 2008).

The discussion of PIN-ABCB interactions invokes mentioning NPA (N-1-Naphthylphthalamic Acid) as one of the most often used polar auxin transport inhibitors. The link between the inhibitor, PINs and ABCBs was noticed during PIN1–ABCB1 and PIN1–ABCB19 co-expression studies, demonstrating a higher degree of NPA sensitivity for those carrier combinations (Blakeslee et al., 2007). Indeed, NPA treatment results in pin-like inflorescences (Gälweiler et al., 1998; Kim et al., 2010), but the direct connection between NPA and PINs has not been demonstrated so far. In this context, it is also worth mentioning, that transport activity of PINs has been shown in a heterologous systems such as yeast and *Xenopus* oocytes (Petrášek et al., 2006; Yang and Murphy, 2009; Zourelidou et al., 2014), that are devoid of ABCBs. This depicts PINs as independent transporters and begs for final clarification of the NPA-PIN-ABCB connection.

## Concluding Remarks

Although PINs play very important roles in plant development, their topology has been only recently experimentally assessed and we still have to wait for NMR or crystallographic data resolving the structure of those carriers in detail. Crystallizing the PIN will be a major effort and a landmark achievement when successful. Yet, before the crystals will be grown and analyzed, we should not lay down our arms and exploit all other possibilities to further structurally and biochemically characterize those important auxin efflux carriers. Over the years substantial knowledge has accumulated on the motifs and amino acids being essential in proper PIN function. Mapping those, and assessing their significance, can be very helpful in the preparation of new PIN constructs, when one knows where to insert and where not to insert a particular tag, which flexible domain to stabilize and which one permits sequence changes. Even partial structural characterization of fragments of the protein will enrich our understanding of the PINs, and support the future expression, purification and structure determination strategies, in which we want to participate, to which we invite and encourage.

## Author Contributions

TN and MZ designed the manuscript outline. TN, MZ, and VB wrote the article. VB and YS prepared the figures. All authors revised the article.

### Conflict of Interest Statement

The authors declare that the research was conducted in the absence of any commercial or financial relationships that could be construed as a potential conflict of interest.
